# Performance of Population Pharmacokinetic Models in Predicting Polymyxin B Exposures

**DOI:** 10.3390/microorganisms8111814

**Published:** 2020-11-18

**Authors:** Vincent H. Tam, Lawrence S. Lee, Tat-Ming Ng, Tze-Peng Lim, Benjamin P. Z. Cherng, Hafeez Adewusi, Kim H. Hee, Ying Ding, Shimin Jasmine Chung, Li-Min Ling, Piotr Chlebicki, Andrea L. H. Kwa, David C. Lye

**Affiliations:** 1 Department of Pharmacy Practice and Translational Research, College of Pharmacy, University of Houston, Houston, TX 77204, USA; adewusih@gmail.com; 2 National Centre for Infectious Diseases, Singapore 308442, Singapore; lawrence_lee@ncid.sg (L.S.L.); Ying_Ding@ncid.sg (Y.D.); Li_Min_Ling@ttsh.com.sg (L.-M.L.); david_lye@ttsh.com.sg (D.C.L.); 3 Tan Tock Seng Hospital, Singapore 308433, Singapore; Tat_Ming_Ng@ttsh.com.sg; 4 Yong Loo Lin School of Medicine, National University of Singapore, Singapore 117597, Singapore; Darylhee@gmail.com; 5 Singapore General Hospital, Singapore 169608, Singapore; lim.tze.peng@sgh.com.sg (T.-P.L.); benjamin.cherng.p.z@singhealth.com.sg (B.P.Z.C.); jasmine.chung.s.m@singhealth.com.sg (S.J.C.); mpchlebicki@gmail.com (P.C.); andrea.kwa.l.h@sgh.com.sg (A.L.H.K.); 6 Duke NUS Medical School, Singapore 169857, Singapore; 7 Lee Kong Chian School of Medicine, Singapore 636921, Singapore

**Keywords:** area under the curve, pharmacokinetics, polymyxins, therapeutic drug monitoring

## Abstract

Polymyxin B is the last line of defense in treating multidrug-resistant gram-negative bacterial infections. Dosing of polymyxin B is currently based on total body weight, and a substantial intersubject variability has been reported. We evaluated the performance of different population pharmacokinetic models to predict polymyxin B exposures observed in individual patients. In a prospective observational study, standard dosing (mean 2.5 mg/kg daily) was administered in 13 adult patients. Serial blood samples were obtained at steady state, and plasma polymyxin B concentrations were determined by a validated liquid chromatography tandem mass spectrometry (LC-MS/MS) method. The best-fit estimates of clearance and daily doses were used to derive the observed area under the curve (AUC) in concentration–time profiles. For comparison, 5 different population pharmacokinetic models of polymyxin B were conditioned using patient-specific dosing and demographic (if applicable) variables to predict polymyxin B AUC of the same patient. The predictive performance of the models was assessed by the coefficient of correlation, bias, and precision. The correlations between observed and predicted AUC in all 5 models examined were poor (r^2^ < 0.2). Nonetheless, the models were reasonable in capturing AUC variability in the patient population. Therapeutic drug monitoring currently remains the only viable approach to individualized dosing.

## 1. Introduction

Over the past decade, a rapid rise in the prevalence of multidrug-resistant bacteria has rendered many first-line antibiotics ineffective, which has also been associated with increased morbidity and mortality [1,2]. Polymyxin B has been used as the last line of defense in treating multidrug-resistant gram-negative bacterial infections. Dosing of polymyxin B is currently based on total body weight, and a substantial intersubject variability can be expected [3]. However, a high prevalence of nephrotoxicity (up to 60%) has also been reported when therapeutic doses of polymyxin B are given [4,5,6]. The lack of a commercial assay makes customized dosing in real time a clinical challenge. Therefore, a more reliable dosing guide would be desirable to the advance medical care of our patients. 

Population pharmacokinetic modeling has been commonly used to characterize drug disposition in general or specific patient cohorts. In addition to point estimates of parameters of interest, the variability (i.e., dispersion) and distribution (i.e., shape) of the pharmacokinetic parameters are also explicitly examined. Moreover, the correlations of selected parameters (e.g., clearance) to common demographic variables (e.g., age, gender, body weight, and renal function) are often explored to further explain the observed variability in a population. If a significant relationship exists, a pharmacokinetic parameter can be expressed as a function of these demographic variables (i.e., covariates). This would allow more accurate prediction of drug exposure to facilitate customized dosing, given that key demographics are available for an individual patient. To explore the feasibility of customized dosing without real-time drug concentration measurements, the objective of the study was to evaluate the performance of different population pharmacokinetic models in predicting polymyxin B exposures observed in individual patients. If a reliable model is identified, it can also be used as the Bayes prior for maximum a posteriori probability (MAP) Bayesian estimation. 

## 2. Materials and Methods 

### 2.1. Study Design, Sites, and Patient Selection

This was a prospective observational study. The study was conducted from March 2016 to March 2018 at two major teaching hospitals in Singapore: Tan Tock Seng Hospital (TTSH) and Singapore General Hospital (SGH). Adult patients (21 years of age or greater) who received intravenous polymyxin B (USP) for the treatment of suspected or confirmed bacterial infections were enrolled. The dose, dosing interval, and infusion duration of polymyxin B administration were as prescribed by the respective attending medical teams. Patients who were pregnant, on dialysis, with burns or spinal cord injury were excluded. 

### 2.2. Pharmacokinetic Assessment

For each subject, four blood samples were obtained serially over one dosing interval at steady state (presumably after the third day of therapy). Since the dosing regimens were not standardized, a sampling scheme spanning the samples over the dosing interval was adopted to accommodate different scenarios. Sample 1 was obtained within 0.5 h prior to the next scheduled dose, sample 2 was obtained 0.5–1 h after the end of drug administration, sample 3 was obtained 3–8 h after the end of drug administration, and sample 4 was obtained within 4 h prior to the next scheduled dose. All samples were specifically timed in relation to drug administration. Blood samples were centrifuged to obtained plasma and stored at −70 °C until analysis. Polymyxin B concentrations in plasma samples were determined using a validated liquid chromatography tandem mass spectrometry method [7]. The intra- and inter-day assay precision (CV%) were reported to be <5.1%. The 4 most abundant polymyxin B components (i.e., polymyxins B1, B2, and B3 and isoleucine B1) were assayed individually, summed up, and reported as the total polymyxin B concentration [8]. The concentration–time profiles observed were characterized using different pharmacokinetic structural models (i.e., one- and two-compartment models with zero-order infusion input) using the ADAPT 5 software (University of Southern California). The weighted least squares (WLS) estimation option was used. We assumed a random error around the observations, and the parameters in the variance model was not estimated. For each subject, the best-fit estimates of total clearance (CL) were used to derive the observed area under the concentration–time profile over 24 h (area under the curve (AUC) = daily dose/CL).

### 2.3. Predictive Performance 

Population pharmacokinetic models of polymyxin B published within the last 10 years were retrieved from the literature. The models were conditioned using patient-specific dosing and demographic (if applicable) variables to predict polymyxin B AUC at steady state (Table A1). Creatinine clearance was estimated using the Crockcroft–Gault formula. For consistent comparison, the correlation between observed and predicted AUC for each individual patient was determined using the same structural model. The predictive performance of the models was assessed at the individual level by the coefficient of correlation, % bias ((predicted AUC − observed AUC) × 100/observed AUC) and % precision (ǀ(predicted AUC – observed AUC)ǀ × 100/observed AUC), respectively. To evaluate the dependability of the models in capturing overall drug exposure in a population, the comparisons were repeated using the mean estimate of CL ± one standard deviation (SD). The reliability of the model prediction was assessed by the percentage of observed AUC that fell within the predicted range (i.e., between the 68% upper and lower limits).

### 2.4. Ethical Approval

This study was approved by the Singapore National Healthcare Group Domain Specific Review Board (DSRB/2013/00991) and Singhealth Centralized Institutional Review Board (CIRB/2014/409/F). Written informed consent was obtained from each patient (or their legal representative) prior to study enrollment. 

## 3. Results

### 3.1. Demographics

Thirteen patients (12 male) were enrolled; none had cystic fibrosis. The mean ± SD age and body weight were 51.5 ± 13.4 years and 69.7 ± 20.2 kg, respectively. The mean ± SD of estimated creatinine clearance was 84 ± 47 mL/min. All patients were given polymyxin B every 12 hours. The dose ranged from 50 mg to 100 mg (1.7 to 3.0 mg/kg daily, mean 2.5 mg/kg daily), and each dose was administered over 0.5 to 4 h. 

### 3.2. Pharmacokinetics

**Figure 1:**
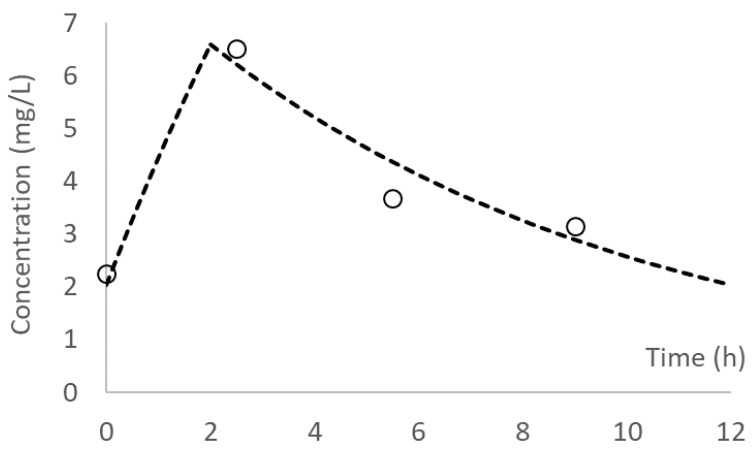
A typical pharmacokinetic profile: open circles depict observed data, and the dashed line represents the best-fit (one compartment) model; r^2^ = 0.93.

The concentration–time profiles were reasonably well characterized by both one-compartment (r^2^ ≥ 0.85) and two-compartment (r^2^ ≥ 0.94) models. Using the one-compartment model, the observed AUC ranged from 47.0 to 135.0 mg.h/L, and the best-fit elimination half-life ranged from 3.4 to 14.8 h (median 6.8 h). A typical fitted pharmacokinetic profile is shown in Figure 1. Using the two-compartment model, the observed AUC ranged from 52.2 to 187.0 mg.h/L.

### 3.3. Predictive Performance 

Five population pharmacokinetic models of polymyxin B were identified, and pertinent characteristics are as shown in Table 1. The estimates of clearance were practically identical in 3 studies (2.4, 2.5., and 2.6 L/h) and no demographic variables were linked to total clearance [9,10,11]. In contrast, body weight was linked to total clearance in a study using a two-compartment model [12]. Finally, creatinine clearance had been linked to total clearance of polymyxin B in cystic fibrosis patients [13]. 

**Figure A1:**
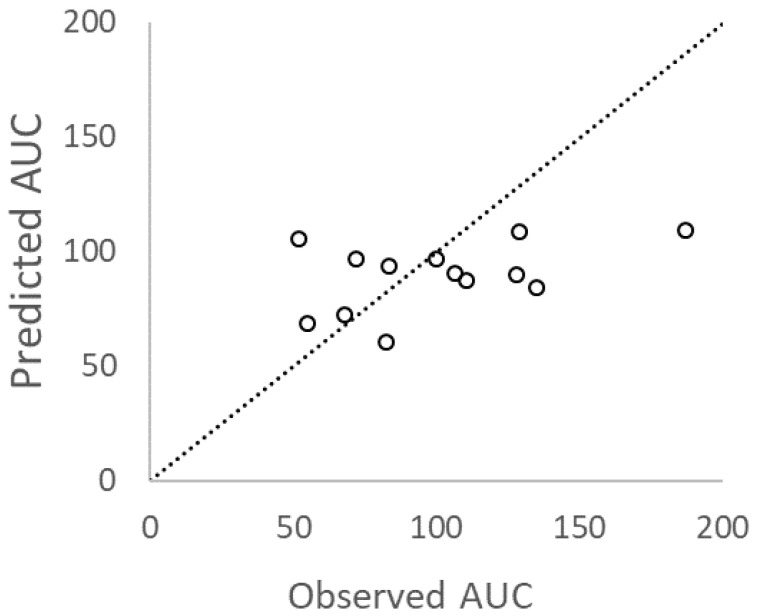
An example of polymyxin B AUC correlation: predictions were based on the Sandri model [12] (highest correlation of the 5 models); r^2^ = 0.197. The dashed line depicts the line of identity, y = x.

At the individual patient level, the correlations between observed and predicted AUC in all 5 models examined were poor (r^2^ < 0.2), regardless of whether demographic variables were incorporated in the prediction (Figure A1). The Sandri model [12] was found to be the least biased, and the Kubin model [10] was the most precise. As shown in Table 1, the intersubject variability was reasonably captured (>75%) by all 5 models using the reported dispersion of total drug clearance.

## 4. Discussion

Polymyxin B is increasingly used clinically, but there is no consensus on its optimal dosing [3]. Bactericidal activity of polymyxin B has been linked to AUC/minimum inhibitory concentration (MIC) ratios [14,15], so it is deemed reasonable to use AUC as a surrogate of dosing adequacy in conjunction with prospective susceptibility data. The pharmacokinetics of polymyxin B has been reported in different patient cohorts. Conventional (weight-based) dosing could be limited as it may not fully account for factors that affect drug exposure. The focus of this study was to explore if prior (published) knowledge of drug behavior could be used to guide customized dosing. As anticipated, there was a significant intersubject variability. Approximately a 3-fold range in AUC was observed with standard dosing. Unfortunately, common demographic variables were not found to be useful in predicting polymyxin B exposures at the individual patient level, regardless of the structural model used (Table A2). This was consistent in 3 out of 5 models evaluated, which did not report a significant covariate of drug clearance. 

The predominant disposition pathway of polymyxin B is not well established. Renal elimination in the unchanged form is unlikely [16,17], and renal metabolism is postulated to be involved (unpublished data). If substantiated, incorporating polymorphism in the drug metabolism could potentially create a framework for correlating predicted polymyxin B exposure and clinical outcomes. A similar approach has been reported in the past using estimated creatinine clearance to predict trough concentrations of cefepime and subsequently correlating drug exposures to clinical success in bacterial pneumonia [18]. 

There were several limitations in our study. Only a limited number of (primarily male) patients were enrolled with standard clinical dosing and dosing frequency. The robustness of our conclusion would be enhanced with a larger sample size and a wider range of dosing regimens. Also, the observed AUC values for comparison were based only 4 data points over a single dosing interval. Given that the expected elimination half-life of polymyxin B ranges from 6–12 h and that the drug is almost always given every 12 hours in the local context, the sampling schedule was deemed to be reasonably informative, balancing various factors such as the logistic feasibility of conducting the study in an acute care setting and therapeutic drug-monitoring practice. More accurate AUC can be estimated with a greater number of samples, but we maintain that our observations are of value to clinician readers managing individual patients. 

## 5. Conclusions

Polymyxin B exposure predicted by the pharmacokinetic models examined did not correlate well to those observed. Further investigations are warranted on how the therapeutic benefit of polymyxin B can be maximized in various patient populations while minimizing toxicity. If available, therapeutic drug monitoring currently remains the only viable approach to individualized dosing.

## Figures and Tables

**Table 1:** Characteristics and summary of population pharmacokinetic models of polymyxin B.

Study Reference	Sandri [12]	Manchandani [9]	Kubin [10]	Avedissian [13]	Miglis [11]
Year published	2013	2017	2018	2018	2018
Sample size	24	35	43	9	52
Compartment model	2	1	1	2	2
% Male	54.2	65.7	70.0	N/A	64.0
Mean/Median age (years)	61.5	58.7	58.0	55.5	47.0
Patient origins	Brazil	U.S., Thailand, Singapore	U.S.	U.S.	U.S.
Patient type	Intensive care	N/A	N/A	Cystic fibrosis	Acutely ill
Clearance covariate	Body weight	None	None	Creatinine clearance	None
Mean % Bias	−0.7	−16.6	−12.0	−5.2	−30.0
Mean % Precision	28.5	22.3	20.5	43.6	31.2
% subject AUC captured *	76.9	84.6	84.6	84.6 **	84.6

Note: * using mean CL ± SD; ** variation around CL_max_; N/A: not available.

## Appendix A

**Table A1:** An example of polymyxin B area under the curve (AUC) prediction by different models.

Model	Sandri	Manchandani	Avedissian
Equation	0.0276 × 75	N/A	(8.65 × (65^7.84^))/((65^7.84^) + (141.24^7.84^)) + 1.43
Predicted clearance (L/h)	2.07	2.5	1.45
Predicted AUC_24_ (mg.h/L)	150/2.07 = 72	150/2.5 = 60	150/1.45 = 103

A 75-kg patient was given polymyxin B 75 mg q12h (daily dose 150 mg), estimated creatinine clearance = 65 mL/min.

**Table A2:** Summary of AUC correlation (r^2^) using different compartment models.

Model	Sandri (2 comp)	Manchandani (1 comp)	Kubin (1 comp)	Avedissian (2 comp)	Miglis (2 comp)
1- Compartment	0.01	0.16	0.16	0.06	0.16
2- Compartment	0.20	0.004	0.004	0.04	0.004

Comp—compartment.
